# Mitochondrial Trafficking of MLKL, Bak/Bax, and Drp1 Is Mediated by RIP1 and ROS which Leads to Decreased Mitochondrial Membrane Integrity during the Hyperglycemic Shift to Necroptosis

**DOI:** 10.3390/ijms24108609

**Published:** 2023-05-11

**Authors:** Matthew A. Deragon, William D. McCaig, Phillip V. Truong, Kevin R. Metz, Katherine A. Carron, Keven J. Hughes, Angeleigh R. Knapp, Molly J. Dougherty, Timothy J. LaRocca

**Affiliations:** Department of Basic and Clinical Sciences, Albany College of Pharmacy and Health Sciences, Albany, NY 12208, USA; matthew.deragon@acphs.edu (M.A.D.);

**Keywords:** necroptosis, apoptosis, hyperglycemia, glucose, MLKL, pores, RIP1, reactive oxygen species

## Abstract

Apoptosis and necroptosis overlap in their initial signaling but diverge to produce non-inflammatory and pro-inflammatory outcomes, respectively. High glucose pushes signaling in favor of necroptosis producing a hyperglycemic shift from apoptosis to necroptosis. This shift depends on receptor-interacting protein 1 (RIP1) and mitochondrial reactive oxygen species (ROS). Here, we show that RIP1, mixed lineage kinase domain-like (MLKL) protein, Bcl-2 agonist/killer (Bak), Bcl-2 associated x (Bax) protein, and dynamin-related protein 1 (Drp1) traffic to the mitochondria in high glucose. RIP1 and MLKL appear in the mitochondria in their activated, phosphorylated states while Drp1 appears in its activated, dephosphorylated state in high glucose. Mitochondrial trafficking is prevented in *rip1* KO cells and upon treatment with N-acetylcysteine. Induction of ROS replicated the mitochondrial trafficking seen in high glucose. MLKL forms high MW oligomers in the outer and inner mitochondrial membranes while Bak and Bax form high MW oligomers in the outer mitochondrial membrane in high glucose, suggesting pore formation. MLKL, Bax, and Drp1 promoted cytochrome *c* release from the mitochondria as well as a decrease in mitochondrial membrane potential in high glucose. These results indicate that mitochondrial trafficking of RIP1, MLKL, Bak, Bax, and Drp1 are key events in the hyperglycemic shift from apoptosis to necroptosis. This is also the first report to show oligomerization of MLKL in the inner and outer mitochondrial membranes and dependence of mitochondrial permeability on MLKL.

## 1. Introduction

Two major forms of mammalian cell death are apoptosis and necroptosis [1,2,3,4]. There is much overlap between both pathways, particularly in the initial steps. Both may be induced by ligation of TNF family cytokines, including TNF-α, Fas ligand (FasL), and TNF-related apoptosis-inducing ligand (TRAIL), to their cognate receptors on the cell surface. Following this, both pathways involve the formation of two death inducing signaling complexes (DISCs), membrane-proximal complex I and cytoplasmic complex II [1,2,3,4]. The formation of complex II can be viewed as a point of divergence between extrinsic apoptosis and necroptosis. When apoptosis is favored, there is proximity-induced activation of caspase-8 in complex II, leading to the cleavage and activation of executioner caspases-3, -6, and -7 [1,4]. The activity of the executioner caspases accounts for cellular changes including DNA and organelle fragmentation, phosphatidylserine externalization, and formation of apoptotic bodies. Macrophages recognize exposed phosphatidylserine on apoptotic cells and bodies, removing them before they lyse, accounting for the non-inflammatory nature of apoptosis [1,4]. When necroptosis is favored, receptor-interacting kinase 1 (RIP1), RIP3, and mixed-lineage kinase domain-like (MLKL) protein are recruited to complex II, forming the necrosome complex [2,3]. Following activation of the necrosome, organelle membranes and the plasma membrane are permeabilized partly due to the pore-forming activity of MLKL [2,5,6]. This leads to cell swelling and lysis concurrent with the release of damage-associated molecular patterns (DAMPs) and other inflammatory mediators accounting for the pro-inflammatory nature of necroptosis [7,8,9]. Like apoptosis, necroptosis occurs in a variety of different cell types, including erythrocytes, leukocytes, cardiac cells, and neurons [10,11,12,13,14,15,16,17].

In addition to MLKL, the effectors downstream of the necrosome include reactive oxygen species (ROS) [7,18,19]. Once activated, the necrosome interacts with metabolic factors in the cells to induce ROS [20]. RIP3 interacts with glycogen phosphorylase and glutamate ammonia ligase which stimulates glycolysis and glutamate metabolism [21]. RIP3 also interacts with pyruvate dehydrogenase and glutamate dehydrogenase in the mitochondria to stimulate the citric acid cycle [21,22]. Activation of these metabolic factors by RIP3 increases respiratory chain activity and the formation of ROS. Respiratory chain activity is further stimulated by RIP1 as it interacts with peroxisome proliferator-activated receptor-gamma coactivator 1 alpha [23]. In addition to their downstream damaging role, ROS are crucial for the oxidation and phosphorylation of RIP1, and thus the activation of necroptosis, during initial signaling steps [17,24]. This creates a positive feedback loop of ROS production and activation of necroptosis [17,24].

Since extrinsic apoptosis and necroptosis share initial steps but then diverge, this suggests that different cellular situations favor one pathway over the other. We have established that high glucose (hyperglycemia) is a cellular situation that favors the activation of TNF-induced necroptosis over apoptosis [25]. The favoring of necroptosis in hyperglycemia accounts for the exacerbated damage during neonatal hypoxia-ischemia (HI) brain injury in this condition [13,17,25]. This hyperglycemic shift from apoptosis to necroptosis depends on RIP1 and mitochondrial ROS [17]. Here, we focus on the trafficking of cell death factors to the mitochondria during the hyperglycemic shift to necroptosis. We show that necrosome proteins traffic from the cytoplasm to the mitochondria during the hyperglycemic shift to necroptosis. The migration of these cell death factors depends on RIP1 as well as ROS. In addition, we show that Bcl-2 agonist/killer (Bak), Bcl-2 associated x (Bax) protein, and MLKL oligomerize in the outer and inner mitochondrial membranes, leading to a loss in mitochondrial membrane potential. The mitochondrial fission regulator, dynamin-related protein 1 (Drp1), is also critical for increased permeability of mitochondrial membranes during the hyperglycemic shift from apoptosis to necroptosis.

## 2. Results

### 2.1. Increased Mitochondrial Trafficking of RIP1, MLKL, Bak, Bax, and Drp1 in Hyperglycemia

**Figure 1:**
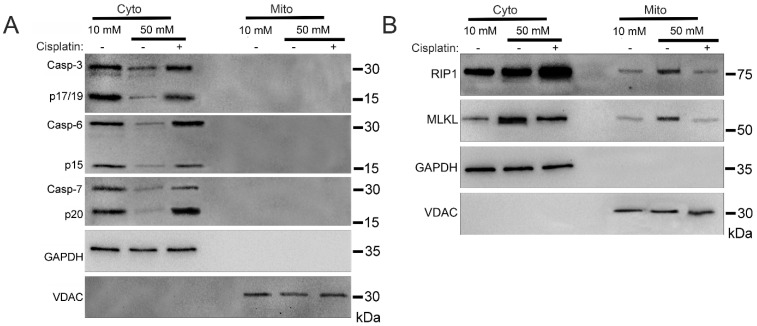
Mitochondrial trafficking of necroptosis factors in high glucose is reversed by induction of apoptosis with cisplatin. U937 cells were grown in media containing 10 or 50 mM glucose for 24 h followed by treatment with 1 ng/mL TNF-α and 250 ng/mL cycloheximide (CHX) with or without the apoptosis inducer, cisplatin, for 2 h at 37 °C. Cells were subjected to mitochondrial fractionation and analyzed via SDS-PAGE and western blot. (**A**) Executioner caspases-3, -6, and -7 do not traffic to mitochondria under either condition. The decrease seen in cytoplasmic levels of caspases in high glucose is reversed by treatment with cisplatin. (**B**) There is increased mitochondrial trafficking of RIP1 and MLKL in 50 mM glucose which is reversed by treatment with cisplatin. Results of three independent experiments. GAPDH = cytoplasm marker, VDAC = mitochondrial marker.

To analyze mitochondrial trafficking of cell death factors during hyperglycemia, we incubated U937 monocytes in 10 or 50 mM glucose for 24 h at 37 °C. Cells were then treated with 1 ng/mL TNF-α combined with 250 ng/mL cycloheximide (CHX) in the presence or absence of apoptosis activator, cisplatin, for 2 h at 37 °C. Following this, we performed cellular fractionation to obtain pure cytoplasmic and mitochondrial fractions [26]. Consistent with previous reports [27], executioner caspases-3, -6, and -7 did not traffic to the mitochondria under any condition (Figure 1A and Figure S1A). Cytoplasmic caspases-3, -6, and -7 all decrease in abundance in high glucose, similar to the decrease in total caspases seen in high glucose previously [17,25]. Downstream activation of apoptosis with cisplatin resulted in a rebounding of cytoplasmic levels of caspases-3, -6, and -7 (Figure 1A and Figure S1A). Necroptosis proteins RIP1 and MLKL exhibited increased mitochondrial trafficking in high glucose which was reversed by cisplatin (Figure 1B and Figure S1B). 

**Figure 2:**
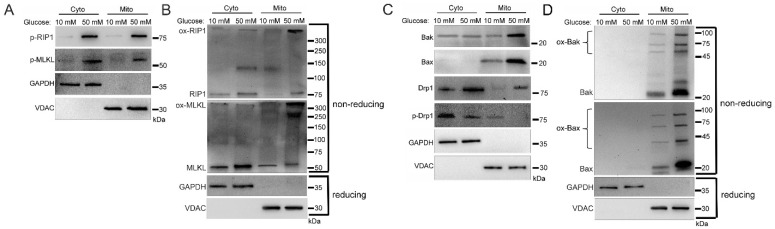
Activation and oligomerization of cell death factors localized in mitochondria during the hyperglycemic shift to necroptosis. U937 cells were grown in media containing 10 or 50 mM glucose for 24 h followed by treatment with 1 ng/mL TNF-α and 250 ng/mL cycloheximide (CHX) for 2 h at 37 °C. Cells were subjected to mitochondrial fractionation and analyzed via SDS-PAGE or non-reducing SDS-PAGE and western blot. (**A**) Phosphorylation of mitochondrial RIP1 and MLKL increases in 50 mM glucose. (**B**) Non-reducing western blot showing that oxidized RIP1 (ox-RIP1) and oxidized MLKL (ox-MLKL) increase in the mitochondria in 50 mM glucose. (**C**) There is increased mitochondrial trafficking of Bak, Bax, and unphosphorylated Drp1 in 50 mM glucose. (**D**) Non-reducing western blot showing that oligomerized Bak (ox-Bak) and Bax (ox-Bax) levels increase in the mitochondrial in 50 mM glucose. Results of three independent experiments. GAPDH = cytoplasm marker, VDAC = mitochondrial marker.

We next determined the activation status of the proteins that were trafficked to the mitochondria. Mitochondrial RIP1 and MLKL increased in phosphorylation in high glucose, indicating their activation [2,3] (Figure 2A and Figure S2A). As determined by non-reducing SDS-PAGE and western blot, mitochondrial RIP1 was oxidized/oligomerized in high glucose (Figure 2B and Figure S2B), which has been shown to be necessary for its activation [17,24]. In addition, oligomerization of MLKL increased in the mitochondria in high glucose (Figure 2B). 

Mitochondrial pore-forming proteins, Bak and Bax [28], also had increased mitochondrial trafficking in high glucose conditions (Figure 2C and Figure S2C). In addition, mitochondrial fission regulator, dynamin related protein 1 (Drp1) [29,30], which is necessary for the mitochondrial recruitment of Bak and Bax [28,31], exhibited increased mitochondrial trafficking in its active, non-phosphorylated form in high glucose (Figure 2C and Figure S2C). Moreover, Bak and Bax increased in their oligomerized forms in the mitochondria in high glucose (Figure 2D and Figure S2D). The formation of oligomers by MLKL, Bak, and Bax suggests pore formation [1,5,6]. 

### 2.2. Cellular Demise by Necroptosis Depends on MLKL, Bax, and Drp1 in High Glucose

**Figure 3:**
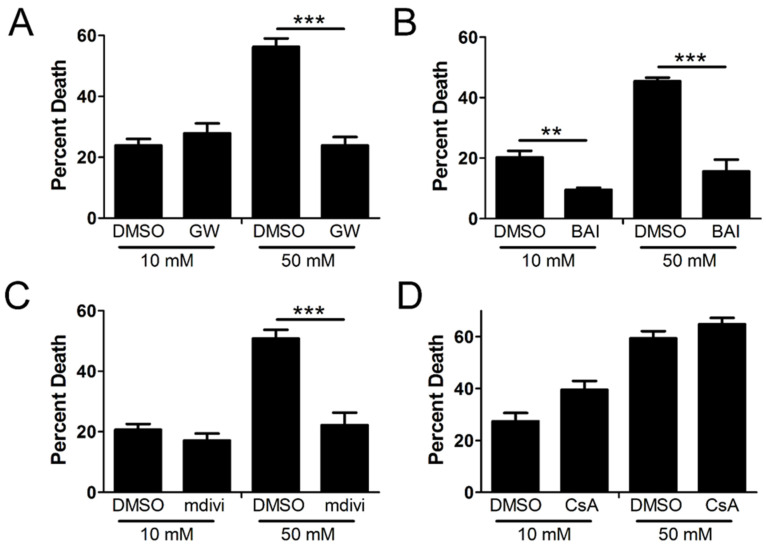
U937 cell death depends on MLKL, Bax, and Drp1 in high glucose. U937 cells were grown in media containing 10 or 50 mM glucose for 24 h followed by treatment with 1 ng/mL TNF-α and 250 ng/mL cycloheximide (CHX) with or without inhibitors for 24 h at 37 °C, followed by WST-1 viability assay. Cell death in 50 mM glucose was prevented by (**A**) MLKL inhibitor, GW806742x (GW), (**B**) Bax inhibitor, BAI, and (**C**) Drp1 inhibitor, mdivi-1. (**D**) CypD inhibitor, cyclosporin A (CsA), which prevents formation of the mitochondrial permeability transition pore (mPTP), has no effect on cell death in either glucose condition. Results of three independent experiments. Two-way ANOVA with Bonferroni posttest. ** *p* < 0.01, *** *p* < 0.001.

Since we confirmed the activation and oligomerization of MLKL, Bak/Bax, and Drp1 in the mitochondria in high glucose, we next asked if the hyperglycemic shift to necroptosis depends on these factors. We incubated U937 monocytes in 10 or 50 mM glucose for 24 h at 37 °C. Cells were then treated with TNF-α combined with 250 ng/mL CHX in the presence or absence of several inhibitors for 24 h at 37 °C followed by WST-1 viability assay. Increased cell death in high glucose was prevented by the MLKL inhibitor, GW806742x (Figure 3A), the Bax inhibitor, BAI (Figure 3B), and the Drp1 inhibitor, mdivi-1 (Figure 3C). Cyclosporin, an inhibitor of CypD and the mitochondrial permeability transition pore (mPTP) [32], failed to prevent cell death in high glucose (Figure 3D). 

### 2.3. Mitochondrial Trafficking in High Glucose Depends on RIP1

**Figure 4:**
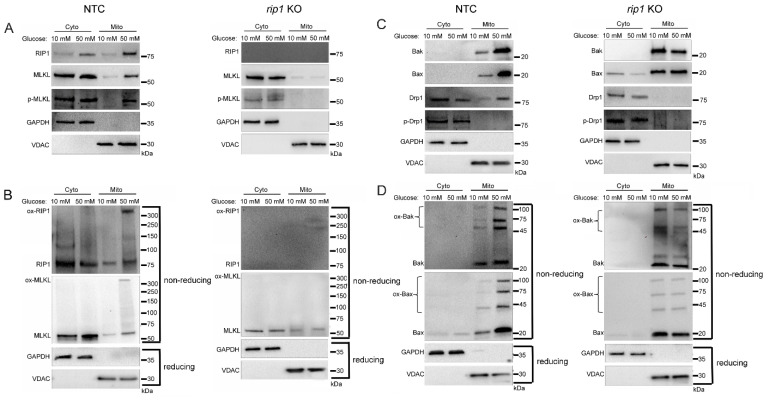
Mitochondrial trafficking of cell death factors in high glucose depends on RIP1. The gene for *rip1* was knocked out in U937 cells using CRISPR-Cas9 or U937 cells were transfected with non-targeting control sgRNA (NTC). NTC and *rip1* KO cells were grown in media containing 10 or 50 mM glucose for 24 h followed by treatment with 1 ng/mL TNF-α and 250 ng/mL cycloheximide (CHX) for 2 h at 37 °C. Cells were subjected to mitochondrial fractionation and analyzed via reducing or non-reducing SDS-PAGE and western blot. (**A**) Phosphorylated MLKL increases in mitochondria in 50 mM glucose in NTC cells but not in *rip1* KO cells. (**B**) Non-reducing western blots showing that oxidized MLKL (ox-MLKL) increases in mitochondria in 50 mM glucose in NTC cells but not in *rip1* KO cells. (**C**) Bak, Bax, and unphosphorylated Drp1 increase in mitochondria in 50 mM glucose in NTC cells but not in *rip1* KO cells. (**D**) Non-reducing western blot showing that mitochondrial levels of oligomerized Bak (ox-Bak) and Bax (ox-Bax) increase in 50 mM glucose in NTC cells but not in *rip1* KO cells. Results of three independent experiments. GAPDH = cytoplasm marker, VDAC = mitochondrial marker.

To explore the role of RIP1 in mitochondrial trafficking of cell death factors in high glucose, we utilized a U937 cell line in which RIP1 had been knocked out using CRISPR-Cas9 (*rip1* KO). Control cells were U937 cells transfected with non-targeting control (NTC) sgRNA. The *rip1* KO and NTC cells were grown in 10 or 50 mM glucose for 24 h at 37 °C. Cells were then treated with 1 ng/mL TNF-α combined with 250 ng/mL CHX for 2 h at 37 °C. Following this, we performed cellular fractionation to obtain pure cytoplasmic and mitochondrial fractions. Similar to WT U937 cells, NTC cells displayed increased mitochondrial trafficking of RIP1, MLKL, and phosphorylated MLKL in high glucose (Figure 4A and Figure S3A). In addition, RIP1 and MLKL were oligomerized in the mitochondria under high glucose conditions (Figure 4B and Figure S3B). Mitochondrial trafficking of these factors did not occur in *rip1* KO cells in high glucose (Figure 4A,B and Figure S3A,B). Furthermore, Bak, Bax, and Drp1 exhibited increased mitochondrial trafficking in NTC cells in high glucose (Figure 4C and Figure S3C). High levels of unphosphorylated Drp1 and oligomerized Bak and Bax were found in the mitochondria under high glucose conditions (Figure 4C,D and Figure S3C,D). Increased levels of these proteins in the mitochondria were prevented in *rip1* KO cells (Figure 4C,D and Figure S3C,D). 

### 2.4. Mitochondrial Trafficking in High Glucose Depends on ROS

**Figure 5:**
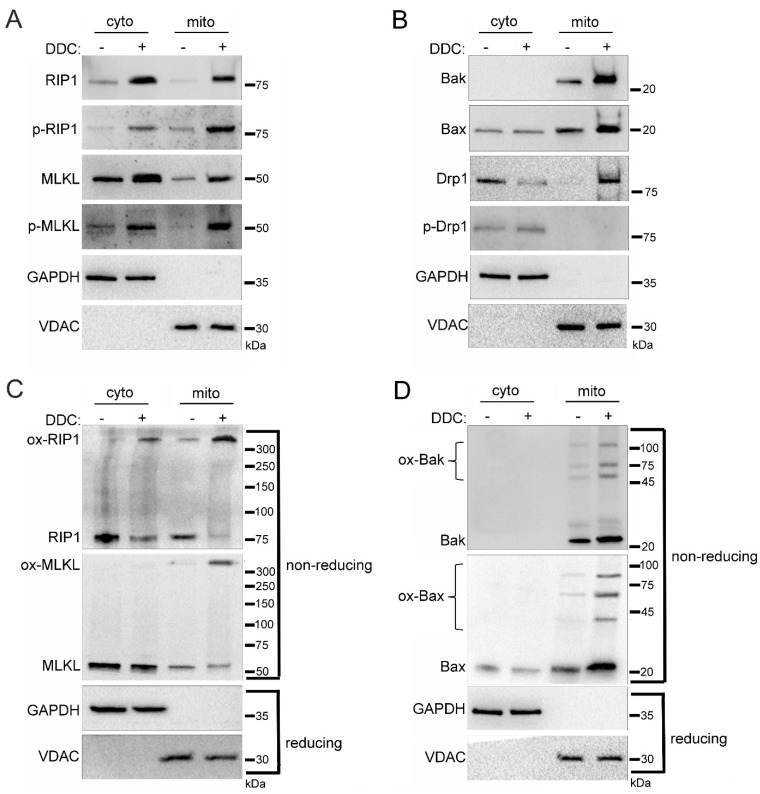
Induction of reactive oxygen species (ROS) in the absence of glucose manipulation replicates mitochondrial trafficking seen in high glucose. U937 cells were grown in media containing 10 mM glucose for 24 h followed by treatment with 1 ng/mL TNF-α and 250 ng/mL cycloheximide (CHX) with or without the superoxide dismutase (SOD) inhibitor, diethyldithiocarbamate (DDC), for 2 h at 37 °C. Cells were subjected to mitochondrial fractionation and analyzed via SDS-PAGE and western blot. There are increased mitochondrial levels of (**A**) phosphorylated RIP1 and MLKL, (**B**) Bak, Bax, and unphosphorylated Drp1, (**C**) oxidized RIP1 and MLKL (ox-RIP1 and ox-MLKL), and (**D**) oligomerized Bak and Bax (ox-Bak and ox-Bax) following DDC treatment. Results of three independent experiments. GAPDH = cytoplasm marker, VDAC = mitochondrial marker.

Previously, we determined that the hyperglycemic shift to necroptosis depends on mitochondrial ROS [17]. Therefore, we investigated the impact of ROS on the mitochondrial trafficking of cell death factors in high glucose conditions. For this, U937 cells were cultured in 10 mM glucose for 24 h at 37 °C. Cells were then treated with 1 ng/mL TNF-α combined with 250 ng/mL CHX in the presence or absence of superoxide dismutase (SOD) inhibitor, diethyldithiocarbamate (DDC), for 2 h at 37 °C. As an inhibitor of SOD [33], we used DDC to induce ROS production without manipulation of glucose levels. Following the 2 h incubation, we performed cellular fractionation to obtain cytoplasmic and mitochondrial fractions. Following treatment with DDC there was a robust increase in levels of RIP1, MLKL, Bak, Bax, and Drp1 in the mitochondria (Figure 5A,B). RIP1 and MLKL that trafficked to the mitochondria during DDC treatment were phosphorylated, indicating their activation [2,3] (Figure 5A and Figure S4A). Mitochondrial Drp1 was unphosphorylated during DDC treatment, indicating its activation as a mitochondrial fission regulator [29,30] (Figure 5B and Figure S4B). Following DDC treatment, the increased mitochondrial levels of RIP1, MLKL, Bak, and Bax were present as high MW oligomers (Figure 5C,D and Figure S4C,D), indicating their activation and suggesting pore formation by MLKL, Bak, and Bax [5,6].

**Figure 6:**
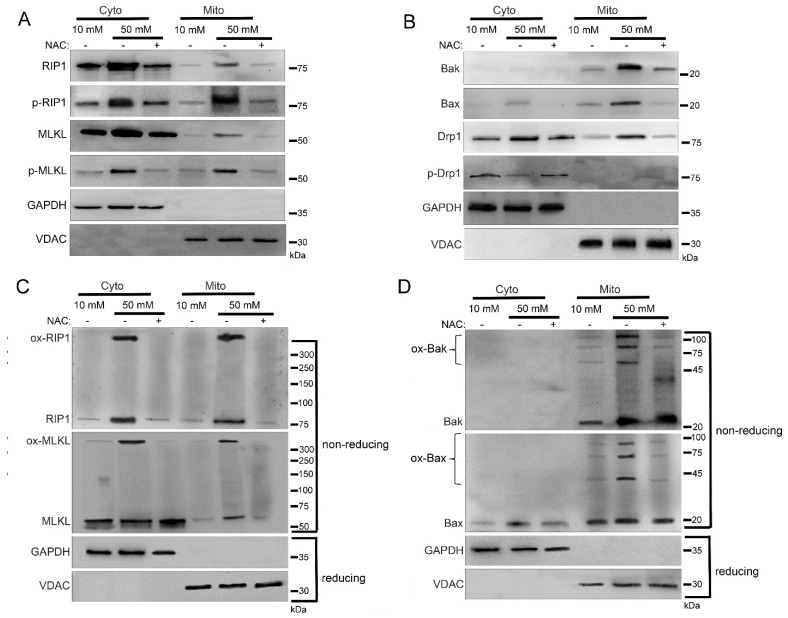
Inhibition of reactive oxygen species (ROS) prevents mitochondrial trafficking in high glucose. U937 cells were grown in media containing 10 or 50 mM glucose for 24 h followed by treatment with 1 ng/mL TNF-α and 250 ng/mL cycloheximide (CHX) with or without the antioxidant, N-acetylcysteine (NAC), for 2 h at 37 °C. Cells were subjected to mitochondrial fractionation and analyzed via SDS-PAGE and western blot. Increased mitochondrial levels of (**A**) phosphorylated RIP1 and MLKL, (**B**) Bak, Bax, and unphosphorylated Drp1, (**C**) oxidized RIP1 (ox-RIP1) and oxidized MLKL (ox-MLKL), and (**D**) oligomerized Bak (ox-Bak) and Bax (ox-Bax) seen in 50 mM glucose are prevented by treatment with NAC. Results of three independent experiments. GAPDH = cytoplasm marker, VDAC = mitochondrial marker.

To further examine the role of ROS in the mitochondrial trafficking of cell death factors during the hyperglycemic shift to necroptosis, we utilized the antioxidant N-acetylcysteine (NAC). For this, we incubated U937 monocytes in 10 or 50 mM glucose for 24 h at 37 °C. Cells were then treated with 1 ng/mL TNF-α combined with 250 ng/mL CHX in the presence or absence of NAC for 2 h at 37 °C followed by cellular fractionation. In agreement with Figure 1, there was an increase in levels of RIP1, MLKL, Bak, Bax, and Drp1 in the mitochondria under hyperglycemic conditions in WT U937 cells (Figure 6A,B and Figure S5A,B). Mitochondrial RIP1 and MLKL were phosphorylated in hyperglycemic conditions while Drp1 was unphosphorylated (Figure 6A,B and Figure S5A,B). However, this mitochondrial trafficking did not occur following NAC treatment in high glucose (Figure 6A,B and Figure S5A,B). Furthermore, oxidized, high MW oligomers of RIP1, MLKL, Bak, and Bax were present in the mitochondria under hyperglycemic conditions, but this was prevented by NAC treatment (Figure 6C,D and Figure S5C,D). 

### 2.5. Sub-Mitochondrial Locations of Cell Death Factors in High Glucose

**Figure 7:**
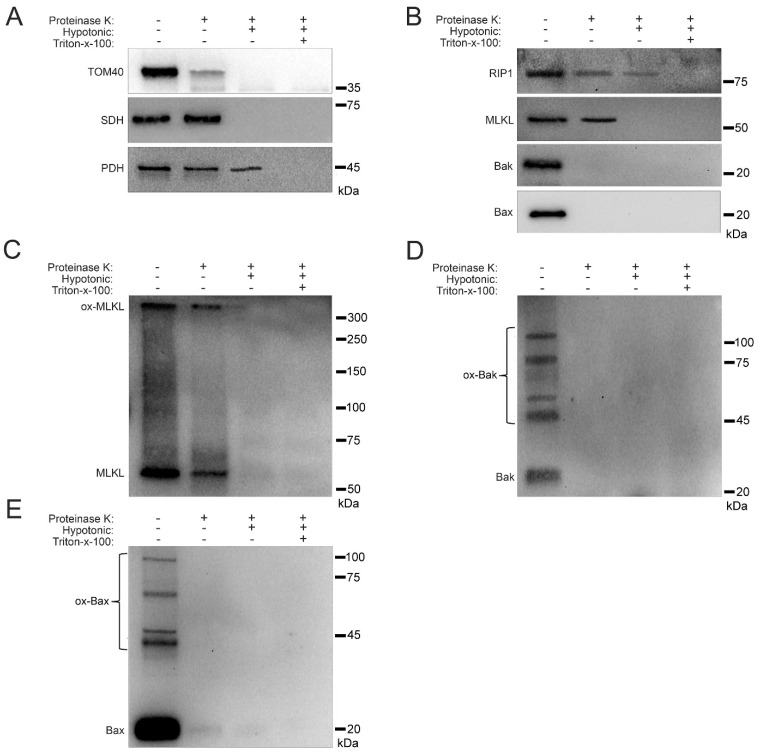
Sub-mitochondrial localization of RIP1, MLKL, Bak, and Bax in high glucose. U937 cells were grown in media containing 10 or 50 mM glucose for 24 h, followed by treatment with 1 ng/mL TNF-α and 250 ng/mL cycloheximide (CHX) for 24 h at 37 °C, followed by mitochondrial fractionation. Isolated mitochondria were treated with proteinase K in different biochemical conditions. (**A**) TOM40, which is known to reside in the outer mitochondrial membrane, is cleaved by proteinase K. Succinate dehydrogenase (SDH), which is known to reside in the inner mitochondrial membrane, is cleaved by proteinase K in hypotonic conditions. Pyruvate dehydrogenase (PDH), which is known to reside in the mitochondrial matrix, is cleaved by proteinase K when in the presence of triton-x-100. (**B**) RIP1 is cleaved by proteinase K in hypotonic conditions as well as in combination with triton-x-100. MLKL is cleaved by proteinase K in normal and hypotonic conditions. Bak and Bax are cleaved by proteinase K in normal conditions. (**C**) Non-reducing western blot showing that oxidized MLKL (ox-MLKL) is cleaved by proteinase K in normal and hypotonic conditions. (**D**) Non-reducing western blot showing that oligomerized Bak (ox-Bak) is cleaved by proteinase K in normal conditions. (**E**) Non-reducing western blot showing that oligomerized Bax (ox-Bax) is cleaved by proteinase K in normal conditions. Results of three independent experiments.

Knowing that RIP1, MLKL, Bak, and Bax show increased trafficking to the mitochondria under hyperglycemic conditions (Figure 1, Figure 2, Figure 4 and Figure 6), we determined the precise mitochondrial location of these proteins. We used a proteinase K cleavage assay under different conditions to determine these locations. U937 cells were cultured in 50 mM glucose for 24 h at 37 °C followed by treatment with 1 ng/mL TNF-α combined with 250 ng/mL CHX for 2 h at 37 °C. After this, cell fractionation was performed to obtain isolated mitochondria. The isolated mitochondria were then exposed to three conditions: (1) proteinase K treatment, (2) proteinase K + hypotonic solution treatment, and (3) proteinase K + hypotonic solution + triton-x-100 treatment. The hypotonic solution lyses the outer mitochondrial membrane giving proteinase K access to proteins in the inner mitochondrial membrane [34]. Triton-x-100 lyses the inner mitochondrial membrane giving proteinase K access to proteins in the mitochondrial matrix [34]. As a proof of principle, we performed western blot for known housekeeping proteins TOM40 (outer membrane protein [35]), succinate dehydrogenase (SDH; inner membrane protein [36]), and pyruvate dehydrogenase (PDH; matrix protein [37]). Upon proteinase K treatment, there was a significant decrease in the amount of TOM40, confirming its location in the outer mitochondrial membrane (Figure 7A and Figure S6A). Following treatment with proteinase K and hypotonic solution, SDH disappeared, confirming its location in the inner mitochondrial membrane (Figure 7A and Figure S6A). After treatment with proteinase K and triton-x-100, PDH disappeared, confirming its location in the mitochondrial matrix (Figure 7A and Figure S6A). RIP1 sequentially decreased in each treatment condition, suggesting that it is present in the outer and inner mitochondrial membranes as well as the mitochondrial matrix (Figure 7B and Figure S6B). MLKL decreased following proteinase K treatment and disappeared following treatment with proteinase K and hypotonic solution, suggesting its presence in both the inner and outer mitochondrial membranes (Figure 7B and Figure S6B). Both Bak and Bax disappeared after treatment with proteinase K, suggesting their presences in the outer mitochondrial membrane (Figure 7B and Figure S6B). Moreover, MLKL existed as an oxidized, high MW oligomer in the outer and inner mitochondrial membranes, while Bak and Bax existed as high MW oligomers in the outer mitochondrial membrane (Figure 7C–E and Figure S6C). This is significant, as it shows oligomerization of MLKL in membranes other than the plasma membrane. In addition, oligomerization of Bak and Bax in the outer mitochondrial membrane is significant, as it demonstrates a potential role for these proteins, normally associated with apoptosis, in mitochondrial permeability in necroptosis. 

### 2.6. Mitochondrial Permeability and Loss of Mitochondrial Membrane Potential (Δψ_m_) in High Glucose Depends on MLKL, Bax, and Drp1

**Figure 8:**
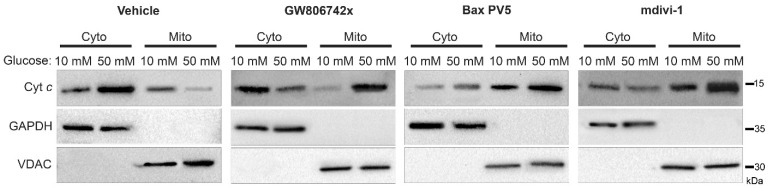
Release of cytochrome *c* from the mitochondria to the cytoplasm in high glucose depends on MLKL, Bax, and Drp1. U937 cells were grown in media containing 10 or 50 mM glucose for 24 h followed by treatment with 1 ng/mL TNF-α and 250 ng/mL cycloheximide (CHX) with or without inhibitors for 2 h at 37 °C. Cells were subjected to mitochondrial fractionation and analyzed via SDS-PAGE and western blot. Cytoplasmic levels of cytochrome *c* increase in 50 mM glucose with a concurrent decrease in mitochondrial levels but this is prevented by MLKL inhibitor, GW806742x, Bax inhibitor, Bax peptide V5 (PV5), and Drp1 inhibitor, mdivi-1. Results of three independent experiments. GAPDH = cytoplasm marker, VDAC = mitochondrial marker.

As we have shown, increased levels of MLKL and Drp1 are activated in the mitochondria during the hyperglycemic shift to necroptosis (Figure 2, Figure 3 and Figure 4 and Figure 6). Moreover, increased levels of Bak and Bax are oligomerized in the outer mitochondrial membrane while increased levels of MLKL are oligomerized in both the outer and inner mitochondrial membranes (Figure 7). Therefore, we wanted to determine the effect of these proteins on mitochondrial permeability during the hyperglycemic shift to necroptosis. We approached this in two ways: (1) measurement of cytochrome *c* (cyt *c*) release to the cytoplasm and (2) measurement of mitochondrial membrane potential (Δψ_m_). For both approaches, U937 cells were cultured in 10 or 50 mM glucose for 24 h at 37 °C. Cells were then treated with 1 ng/mL TNF-α combined with 250 ng/mL CHX in the presence or absence of the following inhibitors and activators: (1) GW806742x (MLKL inhibitor [38]), (2) BAI1 or Bax inhibiting peptide V5 (Bax inhibitors [39,40]), (3) BTSA1 (Bax activator [41]), or (4) mdivi-1 (Drp1 inhibitor [31]). This was followed by either cellular fractionation and western blot (to assess cyt *c* release) or staining with JC-1 dye and fluorescence microscopy (to assess Δψ_m_). In high glucose conditions, there was a robust decrease in mitochondrial levels of cyt *c* concurrent with an increase in its cytoplasmic levels (Figure 8 and Figure S7). 

**Figure 9:**
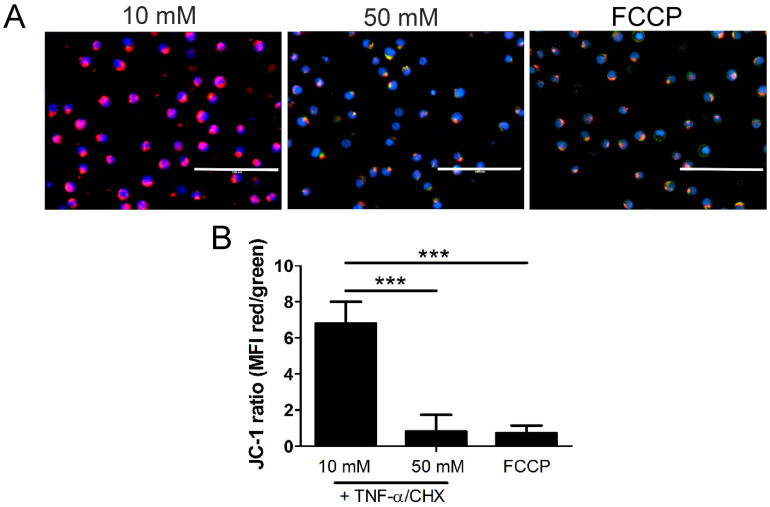
Mitochondrial membrane potential decreases in high glucose. U937 cells were grown in media containing 10 or 50 mM glucose for 24 h followed by treatment with 1 ng/mL TNF-α and 250 ng/mL cycloheximide (CHX) or positive control FCCP for 2 h at 37 °C. Following this, cells were stained with JC-1 mitochondrial membrane potential dye and Hoechst and analyzed via fluorescence microscopy. (**A**) There is a decrease in JC-1 aggregates (red) and an increase in JC-1 monomers (green) in 50 mM glucose, indicating a loss of mitochondrial membrane potential. The increase in green fluorescence appears yellow or orange as it overlaps with the red fluorescence in the images. Scale bars = 100 µm. Blue = Hoechst 33342 nuclear stain. (**B**) Quantification of mean fluorescence intensity (MFI). Results of three independent experiments. Two-way ANOVA with Bonferroni posttest. *** *p* < 0.001.

**Figure 10:**
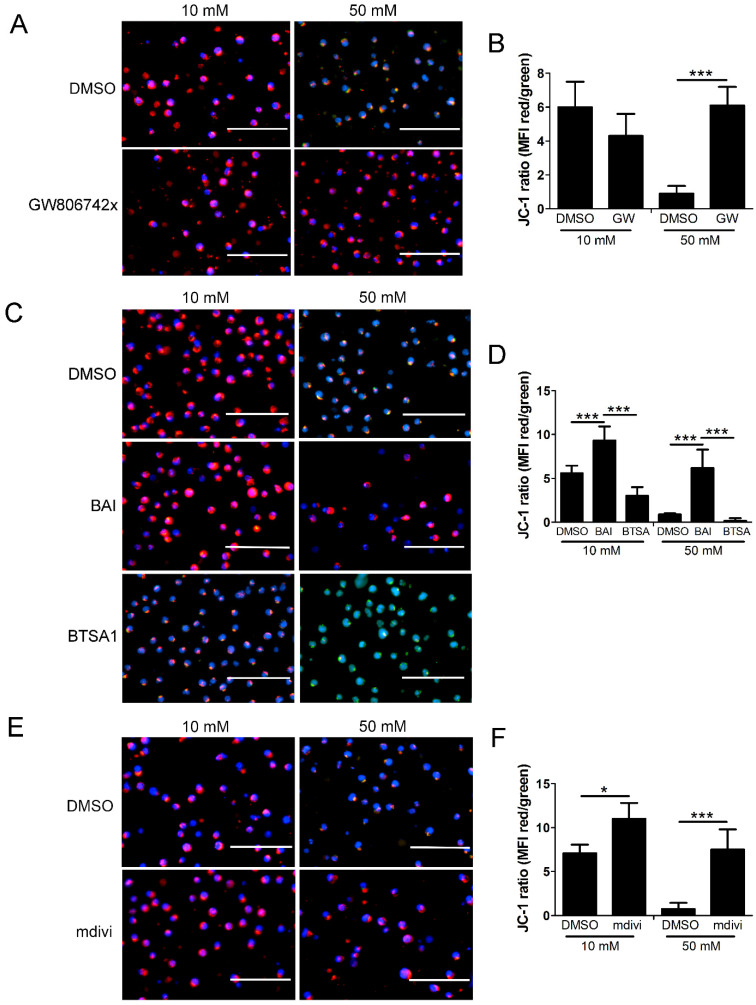
Loss of mitochondrial membrane potential in high glucose depends on MLKL, Bax, and Drp1. U937 cells were grown in media containing 10 or 50 mM glucose for 24 h followed by treatment with 1 ng/mL TNF-α and 250 ng/mL cycloheximide (CHX) for 2 h at 37 °C. Following this, cells were stained with JC-1 mitochondrial membrane potential dye and Hoechst and analyzed via fluorescence microscopy. (**A**) There is a decrease in JC-1 aggregates (red) and an increase in JC-1 monomers (green) in 50 mM glucose, indicating a loss of mitochondrial membrane potential which is prevented by MLKL inhibitor, GW806742x. (**B**) Quantification of mean fluorescence intensity (MFI) of data represented in (**A**). (**C**) Loss of mitochondrial membrane potential is prevented by Bax inhibitor, BAI, and exacerbated by Bax activator, BTSA1. (**D**) Quantification of MFI of data represented in (**C**). (**E**) Loss of mitochondrial membrane potential is prevented by Drp1 inhibitor, mdivi-1. (**F**) Quantification of MFI of data represented in (**E**). In many of the images, the increase in green fluorescence appears yellow or orange as it overlaps with the red fluorescence in the images. Size bars = 100 µm. Blue = Hoechst 33342 nuclear stain. Results of three independent experiments. Two-way ANOVA with Bonferroni posttest. * *p* < 0.05, *** *p* < 0.001.

All three inhibitors prevented the decrease in mitochondrial levels of cyt *c* and the increase in its cytoplasmic levels, suggesting that MLKL, Bax, and Drp1 all contribute to increased mitochondrial permeability during the hyperglycemic shift to necroptosis (Figure 8 and Figure S7). Following JC-1 staining, there was a decrease in JC-1 aggregates and an increase in JC-1 monomers in high glucose conditions, indicating a loss of Δψ_m_ (Figure 9). This loss in Δψ_m_ was prevented by all three inhibitors (Figure 10), suggesting that MLKL, Bax, and Drp1 all contribute to the loss in Δψ_m_ during the hyperglycemic shift from apoptosis to necroptosis.

## 3. Discussion

### 3.1. Significance of Mitochondrial Trafficking

In this report we have shown that mitochondrial trafficking of RIP1, MLKL, Bak, Bax, and Drp1 are key events during the hyperglycemic shift from apoptosis to necroptosis. This is logical, as we previously reported that mitochondrial ROS are critical for the hyperglycemic shift to necroptosis [17]. However, the importance of mitochondrial trafficking to necroptosis has been questioned in the past [42]. While observed to participate in necroptosis, many have found mitochondria to be dispensable for this cell death pathway [42]. Despite this, it has been reported that the necrosome traffics to the mitochondria through interaction with PGAM5 [43,44,45]. This leads to the dephosphorylation of Drp1, allowing it to act as a fission regulator which is necessary for necroptosis execution [43,44]. We have obtained similar findings here, as RIP1 and MLKL traffic to the mitochondria (Figure 1, Figure 2, Figure 4, Figure 6 and Figure 7) and Drp1 is dephosphorylated and necessary for increased mitochondrial permeability (Figure 8), decreased Δψ_m_ (Figure 10), and necroptotic cell death (Figure 3) in high glucose. Therefore, this work supports the idea that mitochondria are essential for necroptosis. We cannot rule out, however, that this mitochondrial involvement may be due to the hyperglycemic conditions under which we have performed our analyses. 

The DNA damage-induced apoptosis activator, cisplatin [46], reversed mitochondrial trafficking of RIP1 and MLKL in high glucose (Figure 1). This suggests that while hyperglycemia induces a shift from apoptosis to necroptosis, artificial induction of apoptosis induced by DNA damage can bypass this shift. The *rip1* KO cells did not exhibit increased mitochondrial trafficking of MLKL, Bak, Bax, and Drp1 in high glucose (Figure 4). This indicates that RIP1 is critical for these events, which agrees with our previous report that RIP1 is necessary for the hyperglycemic shift to necroptosis [17]. Induction of ROS promoted increased mitochondrial trafficking of RIP1, MLKL, Bak, Bax, and Drp1, while their inhibition prevented this mitochondrial trafficking in high glucose conditions (Figure 5 and Figure 6). This indicates that ROS are necessary for the mitochondrial trafficking of these proteins. However, we hypothesize that these proteins traffic to the mitochondria to induce the formation and release of ROS. In addition, RIP1 may traffic to the mitochondrial matrix (Figure 7B) in order to become oxidized and activated [24]. In this context, our findings suggest that ROS may be involved in a positive feedback loop promoting necroptosis during hyperglycemia. We have seen evidence of this potential feedback loop before as we have shown that RIP1 controls ROS levels but, conversely, ROS activate RIP1 in high glucose [17].

### 3.2. Mitochondrial Pore Formation

We have shown that the hyperglycemic shift to necroptosis depends on two pore forming factors that interact with the mitochondria: (1) MLKL and (2) Bak/Bax, (Figure 3). MLKL, Bak, and Bax form oligomers in the mitochondria in high glucose conditions (Figure 2, Figure 4, Figure 6 and Figure 7). MLKL oligomers were found to exist in both the outer and inner mitochondrial membranes while Bak and Bax oligomers were found in the outer mitochondrial membrane only (Figure 7). Moreover, release of cyt *c* and decreased Δψ_m_ depended on MLKL and Bax (Figure 8, Figure 9 and Figure 10). These results strongly suggest that MLKL forms pores in the inner and outer mitochondrial membranes while Bak and Bax form pores in the outer mitochondrial membrane during the hyperglycemic shift to necroptosis. MLKL has been reported to traffic to the mitochondria during necroptosis, however, MLKL pore formation in mitochondrial membranes has not been observed previously [43,45,47,48,49,50,51]. Our results show convincingly that MLKL forms pores in both mitochondrial membranes. This MLKL pore formation in the inner mitochondrial membrane agrees with previous findings showing that MLKL forms pores in cardiolipin-containing liposomes [50]. We believe that these MLKL pores along with Bak/Bax pores allow for the release of ROS from the mitochondria to the cytoplasm during the hyperglycemic shift from apoptosis to necroptosis. 

Bak and Bax have previously been reported to participate in necroptosis in two different ways. The first is by modulating the permeability of the outer mitochondrial membrane in their non-oligomerized state [52,53]. In this scenario, they participate with the mitochondrial permeability transition pore (mPTP) to effect mitochondrial lysis [52,53]. The second way is that Bak and Bax oligomerize in the outer mitochondrial membrane forming pores [47]. These pores then reinforce the opening of the mPTP in the inner mitochondrial membrane, which, in conjunction with Bak and Bax, leads to ROS release into the cytoplasm [47]. In either case, Bak and Bax participate with the mPTP in the inner mitochondrial membrane. In our work, we have ruled out contributions from the mPTP (Figure 3D). Instead, Bak and Bax form pores in the outer mitochondrial membrane, while MLKL appears to form pores in both membranes. Therefore, what we have observed in this report appears to be a previously unknown mechanism. 

### 3.3. Role of Drp1

While it has been reported that Drp1 is not involved in necroptosis [54], there are others who have reported a central role for Drp1 in this cell death pathway [43,44]. When it is involved in necroptosis, Drp1 gets activated by PGAM5S through dephosphorylation [43,44]. Following this, Drp1 causes mitochondrial fission, which may be a mechanism by which ROS are released into the cytoplasm [44]. We have observed that Drp1 traffics to the mitochondria in its non-phosphorylated state in high glucose conditions (Figure 2, Figure 4 and Figure 6), suggesting its activation as a fission regulator. Moreover, the hyperglycemic shift from apoptosis to necroptosis depends on Drp1 (Figure 3C). This could suggest that the fission activity of Drp1 is necessary for the shift to necroptosis in high glucose. However, Drp1 has been reported to have other activities which promote cell death. During apoptosis, Drp1 recruits and binds to Bax, which promotes its activation and oligomerization in the outer mitochondrial membrane [55,56,57]. This interaction results in the activation of Drp1 as well, leading to mitochondrial fission [55,56,57]. Our work shows a requirement for both Bax and Drp1. This may mean that Bax and Drp1 interact during the hyperglycemic shift to necroptosis as they do in apoptosis. 

## 4. Materials and Methods

### 4.1. Cell Culture and Glucose Treatments

WT U937 cells (ATCC, CRL-1593.2) were used in this study, as well as U937 *rip1* KO cells (generated previously from CRL-1593.2 by CRISPR-Cas9 [25]) and U937 cells transfected with non-targeting control (NTC) sg RNA [25]. All cells were maintained in RPMI 1640 with 10 mM glucose and 10% FBS and grown at 37 °C with 5% CO_2_. The *rip1* KO and NTC cells were grown in the presence of 3 µg/mL puromycin. For experiments, cells were grown in RPMI containing 10 or 50 mM glucose and 10% FBS at 37 °C with 5% CO_2_ for 24 h. Following this, cells were centrifuged and placed in RPMI containing 10 mM glucose and 10% FBS. Cells were then used for: cell fractionation, WST-1 assay, or fluorescence microscopy.

### 4.2. Pharmacologic Inhibitors and Activators

Pharmacological inhibitors and activators were used at the following concentrations: BAI (30 µM), Bax inhibiting peptide V5 (100 µM), BTSA1 (30 µM), mdivi-1 (50 µM), GW806742x (1 µM), DDC (1 μM), and NAC (10 mM). All inhibitors and ROS scavengers were purchased from MedChemExpress (Monmouth Junction, NJ, USA).

### 4.3. Cell Fractionation

Cells were treated with 1 µg/mL TNF-α and 250 ng/mL cycloheximide for 2 h at 37 °C. In cases where inhibitors or activators were used, they were added at the same time as TNF-α and CHX. Following treatment, cells were subjected to our isopycnic density gradient fractionation [26]. For full details on this procedure please refer to [26]. Briefly, 6 × 10^8^ U937 cells were centrifuged at 400× *g* for 10 min at room temperature. After washing in PBS, the cells were treated with 15 mL of buffer containing 250 µg/mL digitonin, 50 mM HEPES, and 150 mM NaCl for 20 min on an end-over-end mixer at 4 °C. Following this, cells were centrifuged and the supernatant (crude cytoplasmic fraction) was saved. The crude cytoplasmic fraction was centrifuged at 20,000× *g* for 10 min at 4 °C to remove contaminants from cytoplasm. The pellet from the digitonin treatment was resuspended in 5 mL of buffer containing 20 mM HEPES, 10 mM KCl, 2 mM MgCl_2_, 1 mM EDTA, 1 mM EGTA, 200 mM mannitol, and 70 mM sucrose. This cell solution was then diluted 1:15 in the same buffer and 30 g of 4.8 mm stainless steel beads were added. Cells were homogenized and lysed in a Bullet Blender model 50 DX (Next Advance, Troy, NY, USA) on speed 8 for 5 min at 4 °C. Crude lysates were then sequentially centrifuged at 500, 1000, and 2000× *g* for 10 min at 4 °C to remove cellular debris. Supernatants were then centrifuged at 4000× *g* for 20 min at 4 °C to obtain crude mitochondrial pellets. Crude mitochondrial pellets were resuspended in 2 mL of 45% iodixanol (Axis-Shield, Dundee, Scotland) and loaded at the bottom of a discontinuous density gradient containing 35, 30, 25, 15, and 10% iodixanol. This iodixanol gradient was centrifuged at 100,000× *g* for 18 h at 4 °C to obtain the pure mitochondrial fraction. 

### 4.4. SDS-PAGE and Western Blot

Cytoplasmic and mitochondrial fractions were analyzed by SDS-PAGE and western blot. Next, 4–20% gradient mini-PROTEAN TGX stain-free protein gels (Bio-Rad, Hercules, CA, USA) were run in a Tris/glycine buffered system. For non-reducing SDS-PAGE, β-mercaptoethanol was not included. Samples from SDS-PAGE gels were transferred to PVDF membrane (Millipore-Sigma, Burlington, MA, USA) and were blocked TBS with 0.1% tween-20 containing either 5% milk or BSA (for phosphorylated proteins) for 30 min at room temperature. Blots were probed with primary mAbs for 24 h at 4 °C at the following concentrations: (1) anti-GAPDH (1:10,000), (2) anti-VDAC (1:1000), (3) anti-caspase-3 (1:1000), (4) anti-caspase-6 (1:1000), (5) anti-caspase-7 (1:1000), (6) anti-RIP1 (1:1000), (7) anti-phospho-RIP1 (1:1000), (8) anti-MLKL (1:1000), anti-phospho-MLKL (1:1000), (9) anti-Drp1 (1:1000), (10) anti-phospho-Drp1 (1:1000), (11) anti-Bak (1:1000), (12) anti-Bax (1:1000), (13) anti-cytochrome *c* (1:1000), (14) anti-TOM40 (1:1000), (15) anti-succinate dehydrogenase (1:1000), and (16) anti-pyruvate dehydrogenase (1:1000). All mAbs were purchased from Cell Signaling Technology (Danvers, MA, USA). Anti-rabbit IgG secondary HRP conjugate (ThermoFisher, Waltham, MA, USA) was used at a concentration of 1:5000. After the final wash, blots were developed with chemiluminescent substrate and read in a Bio-Rad ChemiDoc XRS+. All western blots are the results of three independent experiments.

### 4.5. WST-1 Assays

Cells were adjusted to 1 × 10^6^ cells/mL in fresh media and treated with 0.4 lytic units of TNF-α and 250 ng/mL cycloheximide for 24 h. If inhibitors were used, they were added for 1 h prior to the addition of TNF-α and CHX. The lytic units of TNF-α have been described previously [13,17,25]. Pharmacological inhibitors were added where indicated at the concentrations listed above. WST-1 assays were performed in three independent experiments. WST-1 reagent was used to measure cell death according to the manufacturer’s instructions (Takara, Kusatsu, Shiga, Japan). Using WST-1, percent viability was calculated as follows:

% viability = 100 × (absorbance of TNF-/CHX treated cells) ÷ (absorbance of negative control cells.

### 4.6. Proteinase K Assays

Crude mitochondrial pellets were obtained as described above. Mitochondrial pellets were treated with the following conditions for 1 h on ice: (1) 25 µg/mL proteinase K, (2) 25 µg/mL proteinase K + 5 mM Tris-HCl (hypotonic solution), or (3) 25 µg/mL proteinase K + 5 mM Tris-HCl + 0.1% triton-x-100. After incubation, 5 mM PMSF was added for 10 min on ice to stop the proteinase K reaction. Samples were stored at 4 °C. 

### 4.7. Fluorescence Microscopy 

Cells were treated with 1 µg/mL TNF-α and 250 ng/mL cycloheximide for 2 h at 37 °C. In cases where inhibitors or activators were used, they were added at the same time as TNF-α and CHX. Following treatment, cells were prepared for staining by washing off spent media and chemicals from the cells with warm PBS. Cells were spun down at 400× *g* for 10 min and the cell pellets were resuspended in warm RPMI 1640 + 10% FBS containing 2.5 μg/mL Hoechst 33342 (Millipore-Sigma, Burlington, MA, USA) and 100 μL/1 mL from a 1:100 dilution of JC-1 Assay Reagent (Cayman Chemical Company, Ann Arbor, MI, USA). The cells were incubated for 22 min at 37 °C and centrifuged at 400× *g* for 10 min. Cell pellets were resuspended in freshly warmed RPMI 1640 + 10% FBS and diluted 1:1 in a 6-well plate to be imaged on the EVOS FL Digital Inverted Microscope (ThermoFisher, Waltham, MA, USA). Cells were analyzed with the included DAPI, GFP, and RFP fluorescence filters and then presented as overlay images. Fluorescence intensity was measured using ImageJ. 

### 4.8. Statistical Analysis

Two-way ANOVA with Bonferroni posttest was used to analyze the significance of all quantitative data. All results are from three or more independent experiments. Statistics were calculated using GraphPad Prism 5.0.

## Data Availability

Data is contained within the article or supplementary material.

## Supplementary Materials

The following supporting information can be downloaded at: https://www.mdpi.com/article/10.3390/ijms24108609/s1.
