# Supplementary material for: Mitochondrial Trafficking of MLKL, Bak/Bax, and Drp1 Is Mediated by RIP1 and ROS which Leads to Decreased Mitochondrial Membrane Integrity during the Hyperglycemic Shift to Necroptosis
Source: Int J Mol Sci. 2023 May 11;24(10):8609. doi: 10.3390/ijms24108609 (PMC10218403; doi:10.3390/ijms24108609)
Supplement: Supplementary file 1 [file ijms-24-08609-s001.zip › ijms-2341530-supplementary.pdf]

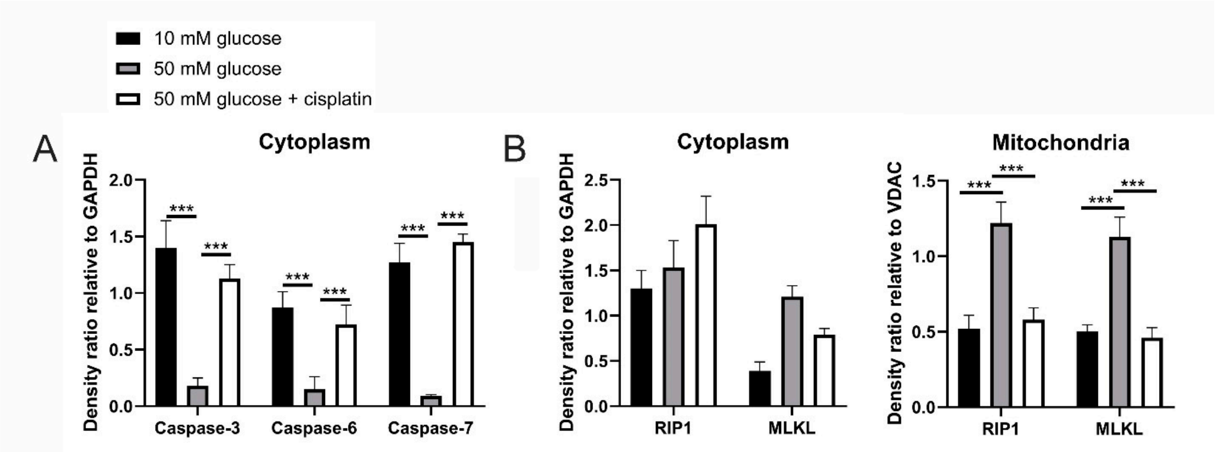

<u>Figure S1</u>: **Cytoplasmic and mitochondrial levels of cell death proteins in 10 and 50 mM glucose with cisplatin. A).** Quantification of cytoplasmic levels of executioner caspases from blot represented in Fig. 1A. Caspases were not detected in the mitochondrial fractions. **B).** Cytoplasmic and mitochondrial levels of RIP1 and MLKL from blot represented in Fig. 1B. Results are from 3 independent experiments. Two-way ANOVA with Bonferroni posttest, ***p<0.001.

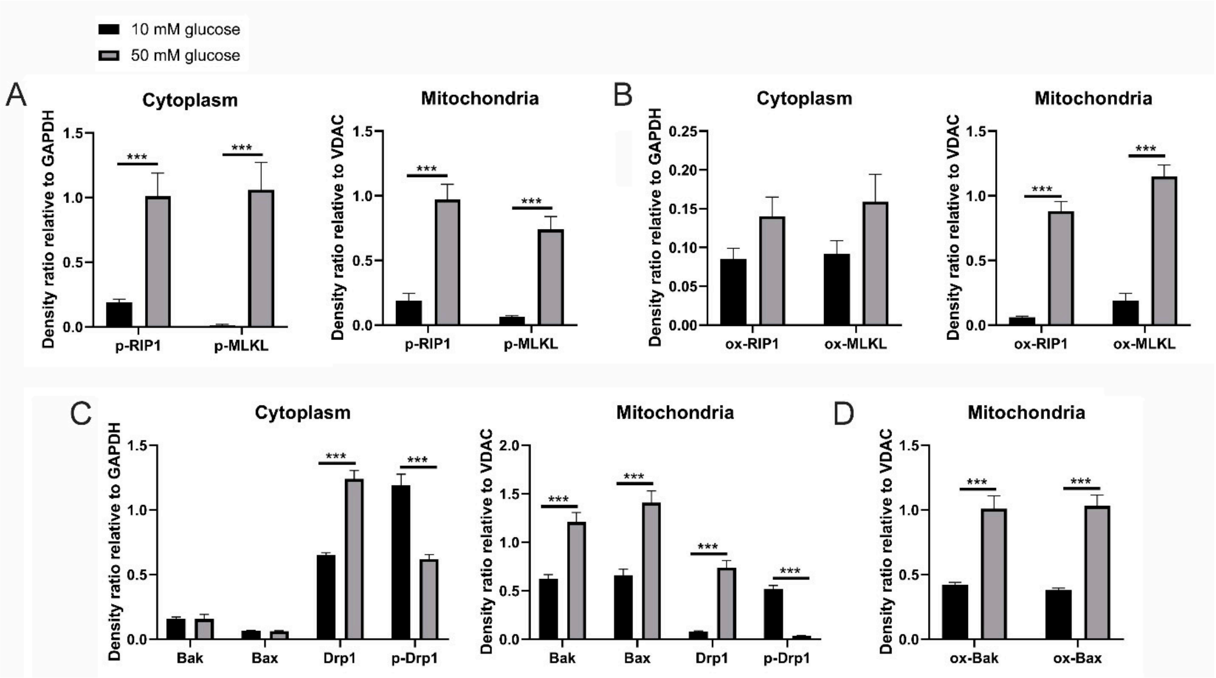

<u>Figure S2</u>: Cytoplasmic and mitochondrial levels of cell death proteins in 10 and 50 mM glucose. A). Quantification of cytoplasmic and mitochondrial levels of phosphorylated RIP1 (p-RIP1) and p-MLKL from blot represented in Fig. 2A. **B).** Cytoplasmic and mitochondrial levels of oxidized RIP1 (ox-RIP1) and ox-MLKL from blot represented in Fig. 2B. **C).** Cytoplasmic and mitochondrial levels of Bak, Bax, Drp1, and p-Drp1 from blot represented in Fig. 2C. **D).** Mitochondrial levels of oxidized Bak (ox-Bak) and ox-Bax from blot represented in Fig. 2D. Oxidized Bak and ox-Bax were not detected in the cytoplasmic fraction. Results are from 3 independent experiments. Two-way ANOVA with Bonferroni posttest, ***p<0.001.

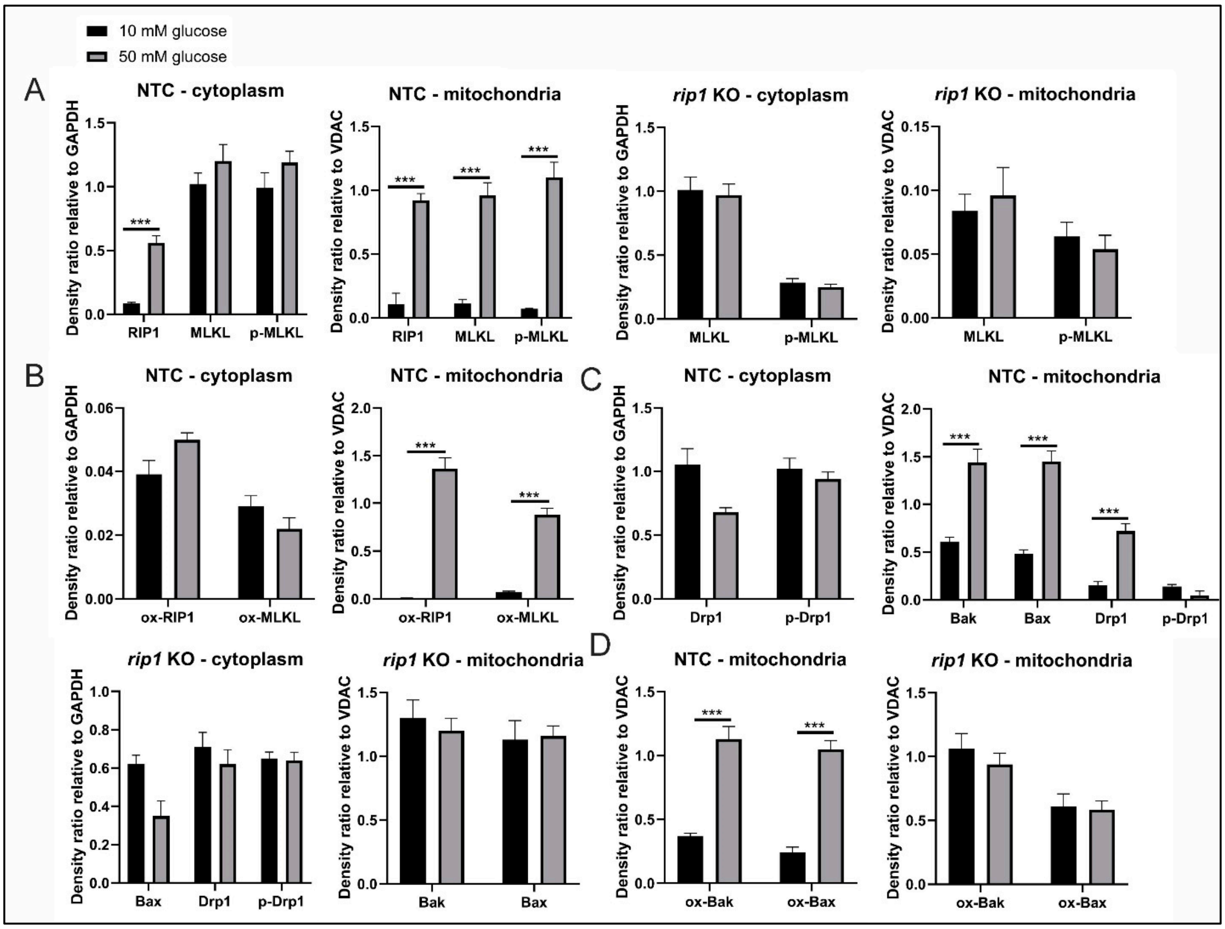

<u>Figure S3</u>: Effect of RIP1 on cytoplasmic and mitochondrial levels of cell death proteins in 10 and 50 mM glucose. A). Quantification of cytoplasmic and mitochondrial levels of RIP1, MLKL and phosphorylated MLKL (p-MLKL) in NTC cells from blots represented in Fig. 4A. RIP1 was not detected in *rip1* KO cells. **B).** Cytoplasmic and mitochondrial levels of oxidized RIP1 (ox-RIP1) and ox-MLKL from blot represented in Fig. 4B. The ox-RIP1 and ox-MLKL were not detected in rip1 KO cells. **C).** Cytoplasmic and mitochondrial levels of Bak, Bax, Drp1, and p-Drp1 from blot represented in Fig. 4C. Bak and Bax were not detected in the cytoplasmic fractions of NTC cells. Bak was not detected in the cytoplasmic fractions of *rip1* KO cells. Drp1 and p-Drp1 were not detected in the mitochondrial fractions of *rip1* KO cells. **D).** Mitochondrial levels of oxidized Bak (ox-Bak) and ox-Bax from blot represented in Fig. 4D. Oxidized Bak and ox-Bax were not detected in the cytoplasmic fractions. Results are from 3 independent experiments. Two-way ANOVA with Bonferroni posttest, ***$p<0.001$.

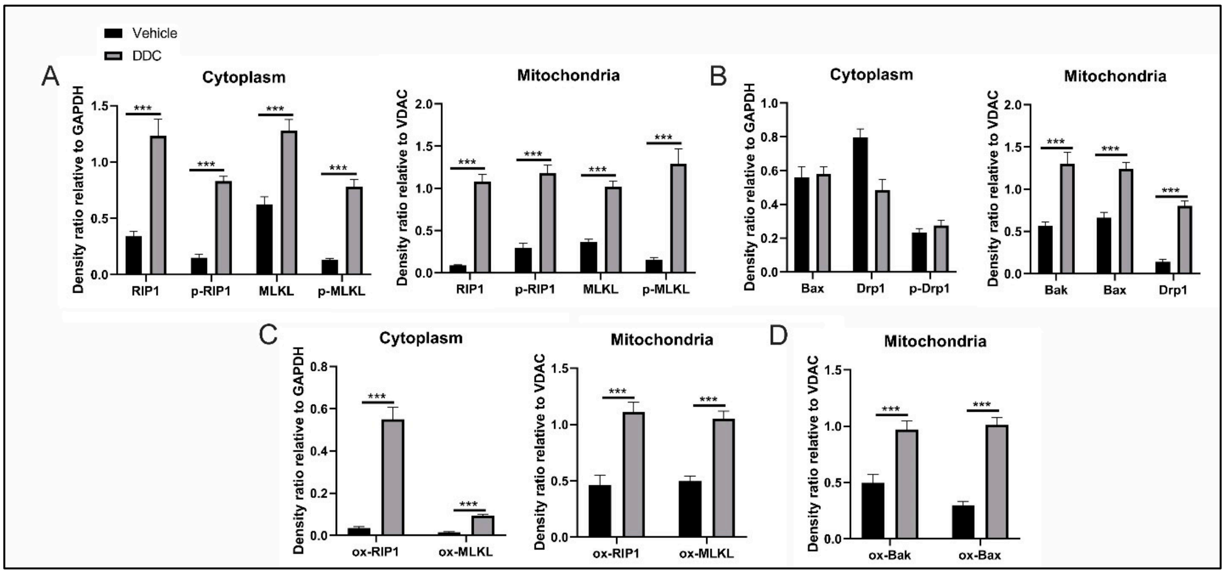

Figure S4: Effect of ROS induction on cytoplasmic and mitochondrial levels of cell death proteins. A). Quantification of cytoplasmic and mitochondrial levels of RIP1, MLKL, phosphorylated RIP1 (p-RIP1), and p-MLKL from blots represented in Fig. 5A. **B).** Cytoplasmic and mitochondrial levels of Bak, Bax, Drp1, and p-Drp1 from blot represented in Fig. 5B. Bak was not detected in the cytoplasmic fractions while p-Drp1 was not detected in the mitochondrial fractions. **C).** Cytoplasmic and mitochondrial levels of oxidized RIP1 (ox-RIP1) and ox-MLKL from blot represented in Fig. 5C. **D).** Mitochondrial levels of ox-Bak and ox-Bax from blot represented in Fig. 4D. Oxidized Bak and ox-Bax were not detected in the cytoplasmic fractions. Results are from 3 independent experiments. Two-way ANOVA with Bonferroni posttest, ***p<0.001.

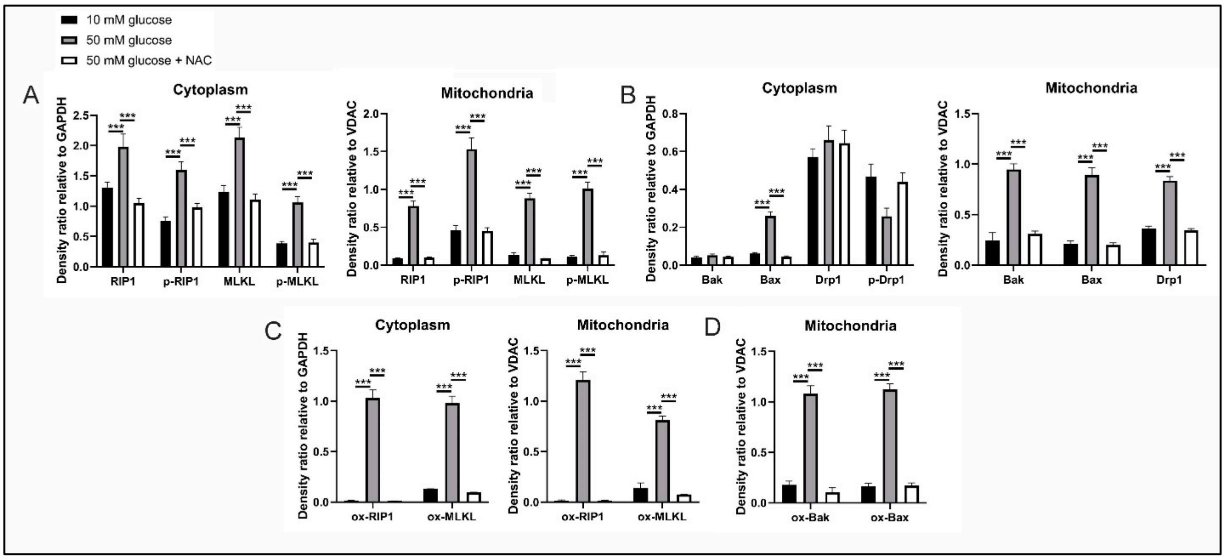

<u>Figure S5</u>: Effect of ROS inhibition on cytoplasmic and mitochondrial levels of cell death proteins in 10 and 50 mM glucose. A). Quantification of cytoplasmic and mitochondrial levels of RIP1, MLKL, phosphorylated RIP1 (p-RIP1), and p-MLKL from blots represented in Fig. 6A. **B).** Cytoplasmic and mitochondrial levels of Bak, Bax, Drp1, and p-Drp1 from blot represented in Fig. 6B. Phosphorylated Drp1 was not detected in mitochondrial fractions. **C).** Cytoplasmic and mitochondrial levels of oxidized RIP1 (ox-RIP1) and ox-MLKL from blot represented in Fig. 6C. **D).** Mitochondrial levels of ox-Bak and ox-Bax from blot represented in Fig. 6D. Oxidized Bak and ox-Bax were not detected in the cytoplasmic fractions. Results are from 3 independent experiments. Two-way ANOVA with Bonferroni posttest, ***p<0.001.

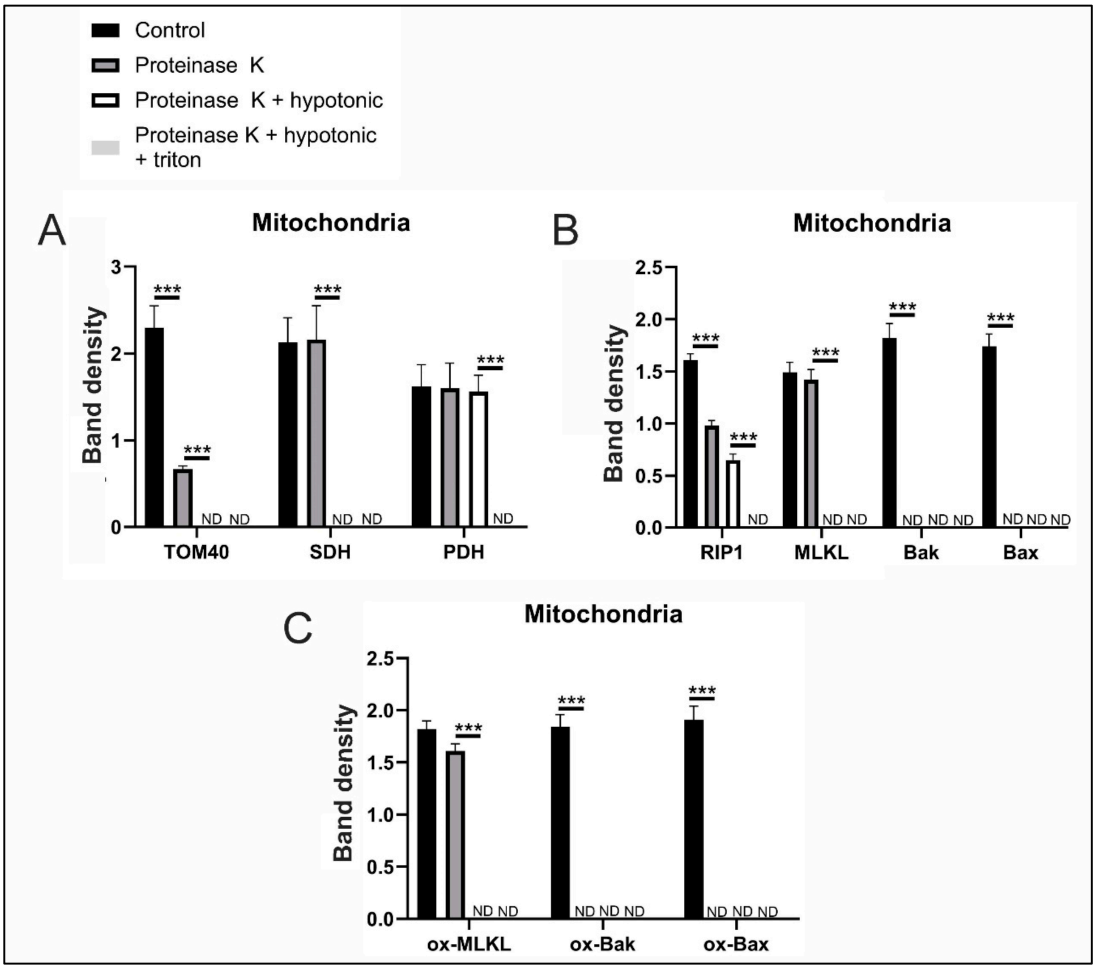

<u>Figure S6</u>: Sub-mitochondrial locations of cell death proteins in 10 and 50 mM glucose. A). Quantification of TOM40, succinate dehydrogenase (SDH) and pyruvate dehydrogenase (PDH) in mitochondrial fractions from blot represented in Fig 7A. **B).** Quantification of RIP1, MLKL, Bak, and Bax in mitochondrial fractions from blot represented in Fig. 7B. **C).** Quantification of oxidized MLKL (ox-MLKL), ox-Bak, and ox-Bax in mitochondrial fractions from blots represented in Figs. 7C-E. ND = not detected. Results are from 3 independent experiments. Two-way ANOVA with Bonferroni posttest, ***p<0.001.

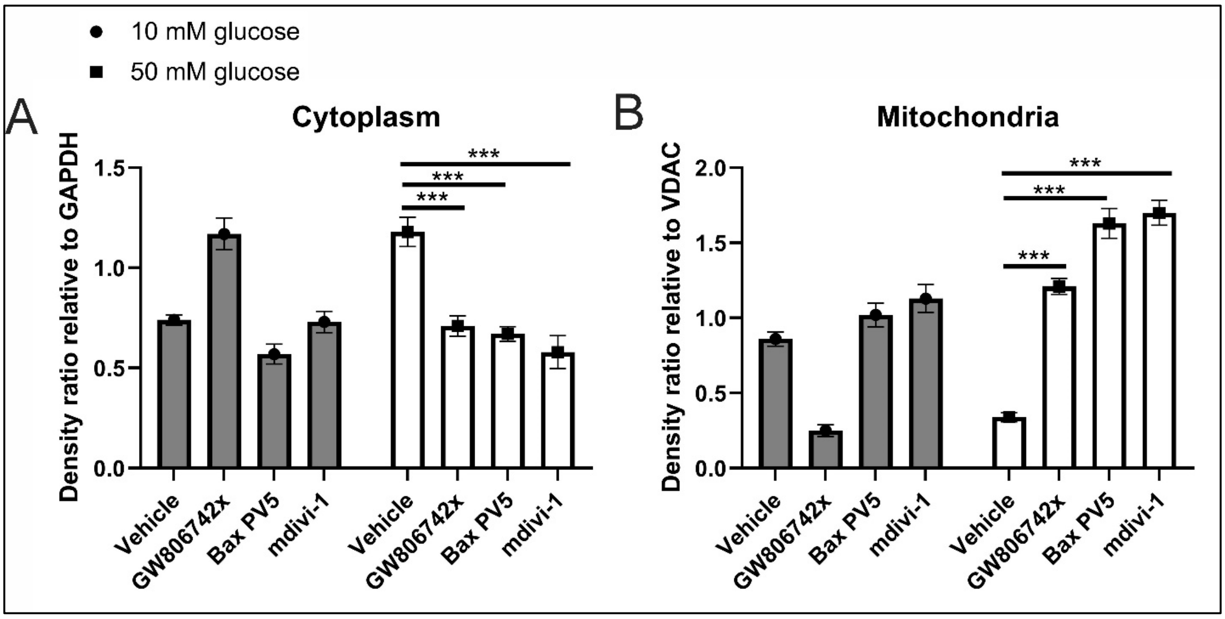

Figure S7: Effect of MLKL, Bax, and Drp1 inhibition on cytoplasmic and mitochondrial levels of cytochrome *c* (cyt *c*). A). Quantification of cyt *c* from cytoplasmic fractions from blots represented in Fig. 8. **B).** Quantificaiton of cyt *c* from cytoplasmic fractions from blot represented in Fig 8. Results are from 3 independent experiments. Two-way ANOVA with Bonferroni posttest, ***p<0.001.
